# Identification of candidate genes encoding tumor-specific neoantigens in early- and late-stage colon adenocarcinoma

**DOI:** 10.18632/aging.202370

**Published:** 2021-01-10

**Authors:** Chong Wang, Wenhua Xue, Haohao Zhang, Yang Fu

**Affiliations:** 1 Department of Hematology, The First Affiliated Hospital of Zhengzhou University, Henan, China; 2 Department of Pharmacy, The First Affiliated Hospital of Zhengzhou University, Henan, China; 3 Department of Endocrinology, The First Affiliated Hospital of Zhengzhou University, Henan, China; 4 Department of Gastrointestinal Surgery, The First Affiliated Hospital of Zhengzhou University, Henan, China

**Keywords:** neoantigens, colon adenocarcinoma, sequencing, recurrent mutations, machine learning

## Abstract

Colon adenocarcinoma (COAD) is one of the most common gastrointestinal malignant tumors and is characterized by a high mortality rate. Here, we integrated whole-exome and RNA sequencing data from The Cancer Genome Atlas and investigated the mutational spectra of COAD-overexpressed genes to define clinically relevant diagnostic/prognostic signatures and to unmask functional relationships with both tumor-infiltrating immune cells and regulatory miRNAs. We identified 24 recurrently mutated genes (frequency > 5%) encoding putative COAD-specific neoantigens. Five of them (*NEB*, *DNAH2*, *ABCA12*, *CENPF* and *CELSR1*) had not been previously reported as COAD biomarkers. Through machine learning-based feature selection, four early-stage-related (*COL11A1*, *TG*, *SOX9*, and *DNAH2*) and four late-stage-related (*COL11A1*, *SOX9*, *TG* and *BRCA2*) candidate neoantigen-encoding genes were selected as diagnostic signatures. They respectively showed 100% and 97% accuracy in predicting early- and late-stage patients, and an 8-gene signature had excellent prognostic performance predicting disease-free survival (DFS) in COAD patients. We also found significant correlations between the 24 candidate neoantigen genes and the abundance and/or activation status of 22 tumor-infiltrating immune cell types and 56 regulatory miRNAs. Our novel neoantigen-based signatures may improve diagnostic and prognostic accuracy and help design targeted immunotherapies for COAD treatment.

## INTRODUCTION

Colorectal cancer (CRC) is the third most common cancer and the second leading cause of cancer-related death in the United States [1]. Colon adenocarcinoma (COAD) is a CRC subtype associated with high mortality [2]. Even though survival prognosis has modestly improved over the last three decades, poor survival and high recurrence still entail a pressing need for novel diagnostic biomarkers and therapeutic targets for COAD [3]. Although chemotherapy shows significant therapeutic value, surgery is still the only curative form of treatment for this CRC form [4].

Immunotherapies that boost the ability of endogenous T cells to destroy cancer cells have shown therapeutic efficacy in various human malignancies [5]. The tumoricidal activity of T cells is basically determined by recognition of immunogenic peptides expressed by cancer cells, termed tumor-specific antigens (TSAs) or neoantigens. Comprehensive analysis of transcriptome and whole exome sequencing (WES) data allows identification of tumor-specific mutations giving rise to neoantigens, which can be eventually selected as diagnostic/prognostic biomarkers and therapeutic targets [6]. To screen candidate neoantigens derived from tumor-specific mutations, we evaluated the expression of the corresponding host genes in both COAD and normal tissues. Only recurrent mutations that were highly expressed in tumor cells and lowly or not expressed in normal cells were selected as potential sources of candidate neoantigens [7].

WES analysis aims to uncover the most frequently mutated genes for a given condition or disease [8]. Nevertheless, multiple genetic variants may be present within individual genes, especially long ones. In tumor suppressor genes, multiple mutations, usually scattered across different loci, may lead to loss-of-function and drive tumorigenesis if both alleles become deficient or inactivated (i.e. the “two-hit” hypothesis). In contrast, mutations in oncogenes are often aggregated, triggering a specific pathogenic function [9, 10]. Therefore, canonical gene-level analysis is not fully adequate to mine disease-specific properties. Instead of selecting genes containing the most mutations, we assessed mutation recurrence among patients at the nucleotide resolution. Subsequently, we integrated RNAseq data to screen recurrent mutations within overexpressed genes in COAD tissues, compared to normal ones. In this manner, we separately compared early and late stage COAD data and contrasted these findings with normal controls to analyze differential gene expression, mutational profiles, and hypothetical functions therefore affected. This approach led us to identify several differentially expressed genes (DEGs) with potential to generate tumor-specific neoantigens. We then addressed the correlations between these DEGs and both regulatory miRNAs and infiltrating tumor cells, and applied a machine learning model to select, among the candidate neoantigen-forming DEGs, molecular signatures for COAD diagnosis and prognosis. Our findings may serve to improve diagnostic and prognostic accuracy in COAD, and help also design targeted immunotherapeutic approaches to increase patient survival.

## RESULTS

### COAD data selection and clinical information

**Figure 1:**
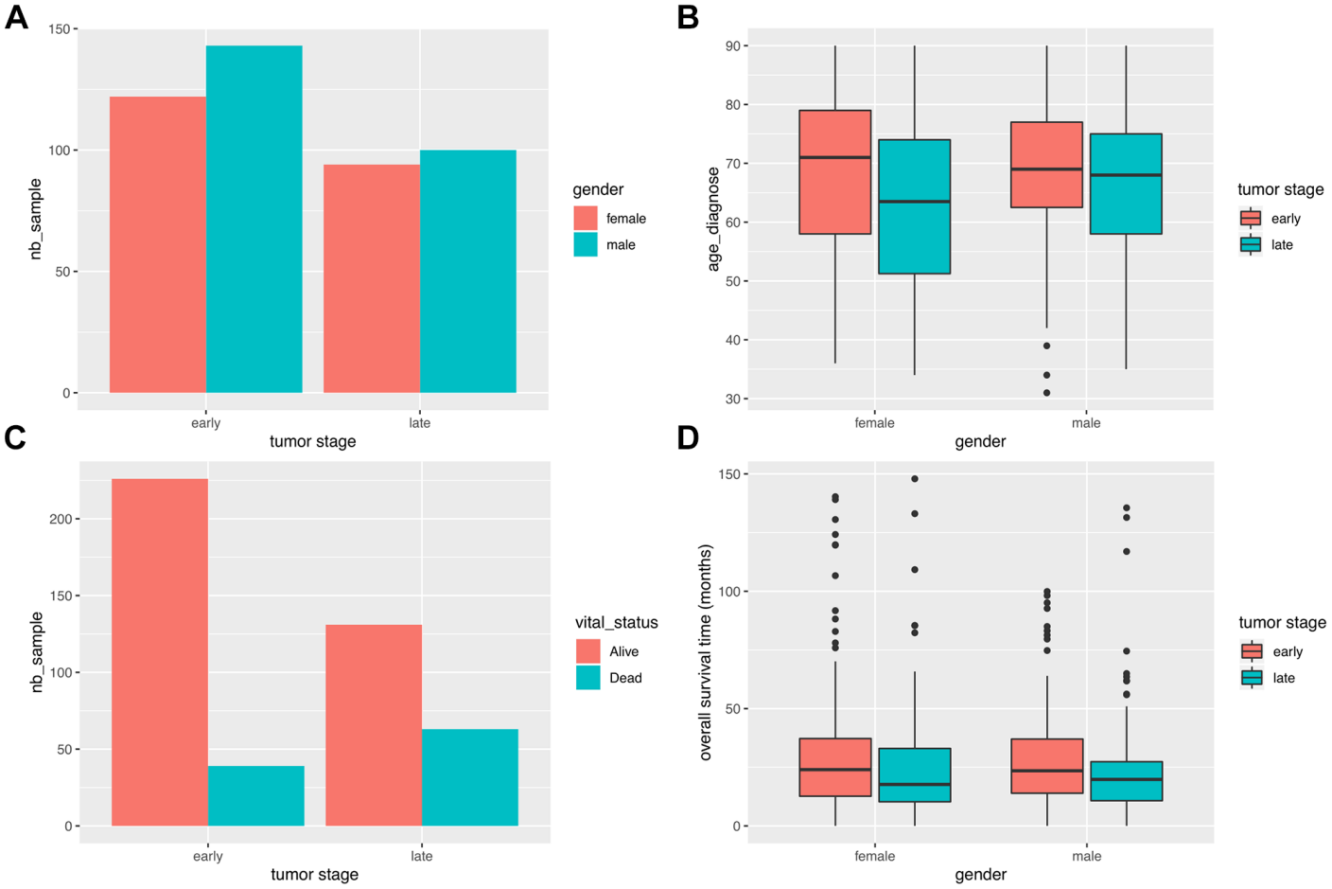
**Clinical characteristics of COAD patients.** (**A**) Stage distribution between male and female COAD patients. (**B**) Gender-based distribution of age of first diagnosis and stage. (**C**) Vital status distribution according to stage. (**D**) Survival time distribution for gender and stage.

We retrieved from TCGA a total of 459 COAD samples, including normal controls with clinical information. Of those, 329 and 399 samples underwent RNAseq and WES analysis, respectively. In addition, miRNA sequencing data was retrieved from 261 samples. A total of 20,529 mRNAs and 2,113 miRNAs were identified in the aggregated sequencing data. Clinical information, including gender, stage, vital status, and survival time are shown in Figure 1.

It can be seen that the number of male patients is slightly higher than the number of female patients at both early and late stages. The average age of first diagnosis shows no difference between genders, although late stage cases are more likely to be diagnosed in younger groups. As expected, the mortality ratio was significantly higher for late stage cases (P = 1.639e-05; Fisher’s exact test). There were no differences in overall survival (OS) time between genders.

### Differential gene expression analysis

**Figure 2:**
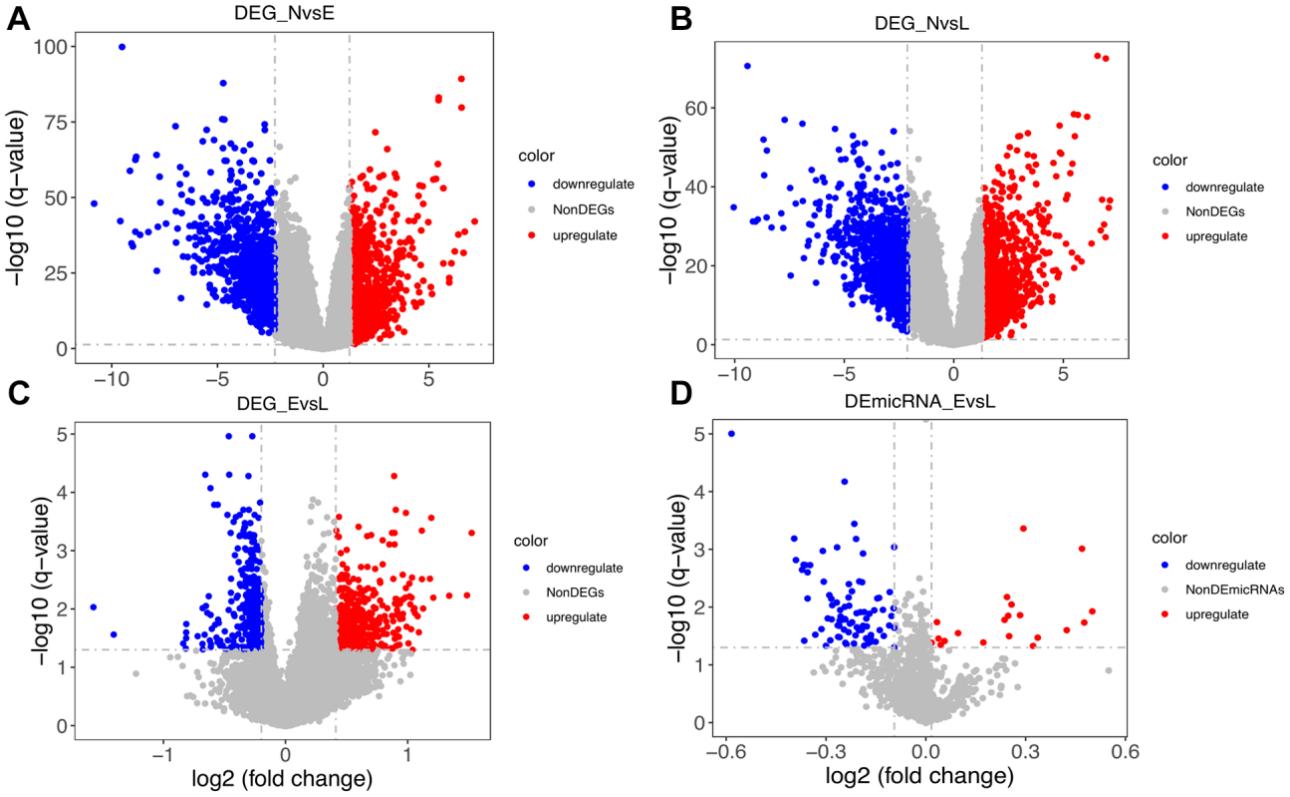
**Distribution of differentially expressed genes.** (**A**) Distribution of DEGs between normal (N) and early-stage (E) samples. (**B**) Distribution of DEGs between N and late-stage (L) samples. (**C**) Distribution of DEGs between E and L samples. (**D**) Distribution of differentially expressed microRNAs between E and L samples.

We conducted differential analysis of mRNA expression profiles between normal vs total, early stage, and late stage tumors, as well as between early vs late stage tumors samples (Table 1 and Figure 2A–2C).

**Table 1:** Number of differentially expressed genes among conditions and stages.

	**N vs. T**	**N vs. E**	**N vs. L**	**E vs. L**
Upregulated	1026	1027	1027	482
Downregulated	1027	1027	1027	369
Low cutoff	-2.21	-2.28	-2.12	-0.2
High cutoff	1.25	1.24	1.29	0.41

N, T, E, and L represent normal, tumor (any stage), early stage, and late stage, respectively. Low and high cutoffs were determined using 95% confidence interval limits across the logFC values of all genes.

As shown in Table 1 and Figure 2A, 2B, a balanced distribution of up- and down-regulated genes was detected, regardless of tumor stage, upon comparison with normal control data. In contrast, most DEGs between early and late stage tumors were upregulated (Figure 2C). Since only 8 normal samples were included in the miRNA data, we only compared miRNA profiles between early and late stage tumors. As shown in Figure 2D, most differentially expressed miRNAs were downregulated. This finding seems to be consistent with the observed DEG pattern, considering that negative, rather than positive, regulation is usually exerted by miRNAs on protein-coding transcripts.

### Gene clustering and functional analysis

**Figure 3:**
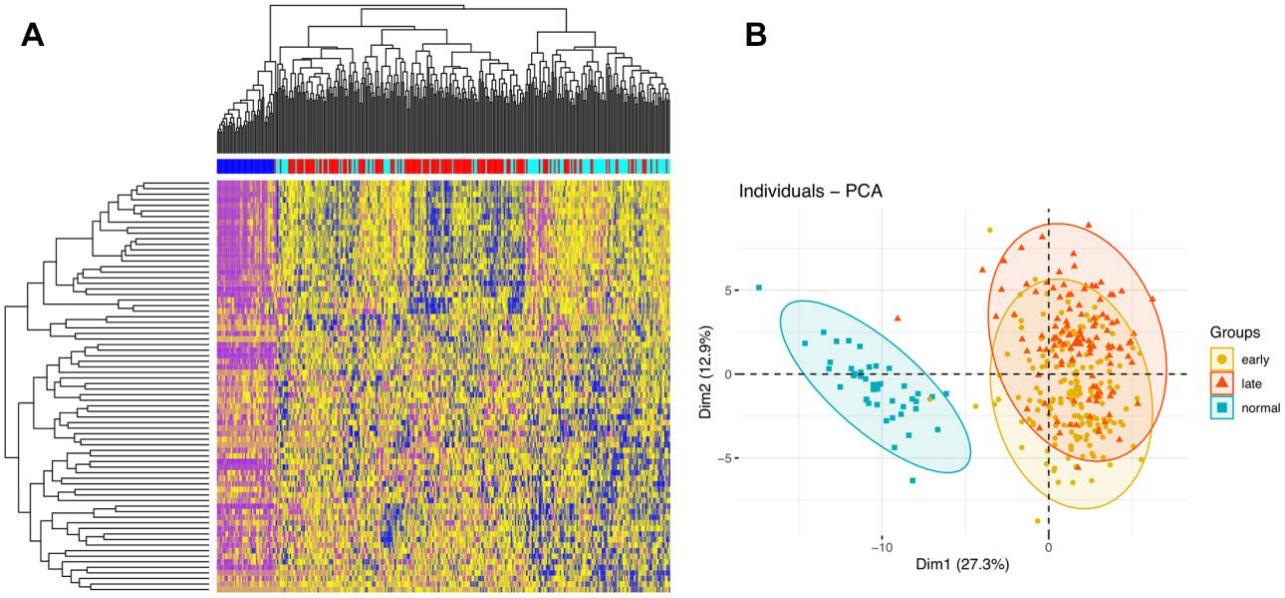
**Clustering analyses.** (**A**) Hierarchical clustering analysis of DEGs. The intersections of DEGs of early vs late stage are used to cluster samples. Normal, early stage, and late stage samples are marked by dark blue, red, and light blue, respectively. (**B**) PCA of samples.

The identified DEGs exhibited diverse expression patterns among normal, early-stage, and late-stage samples. As seen in Figure 3A, tumor and normal samples were separated into different groups based on DEG profiling. To some extent, early and late stage patients also presented some distinctions. Principal component analysis (PCA) was then used to visualize the distribution of all samples based on the first two principal components (Figure 3B). Consistent with the heatmap analysis, the result showed that tumor samples showed diverse patterns compared with normal ones.

**Figure 4:**
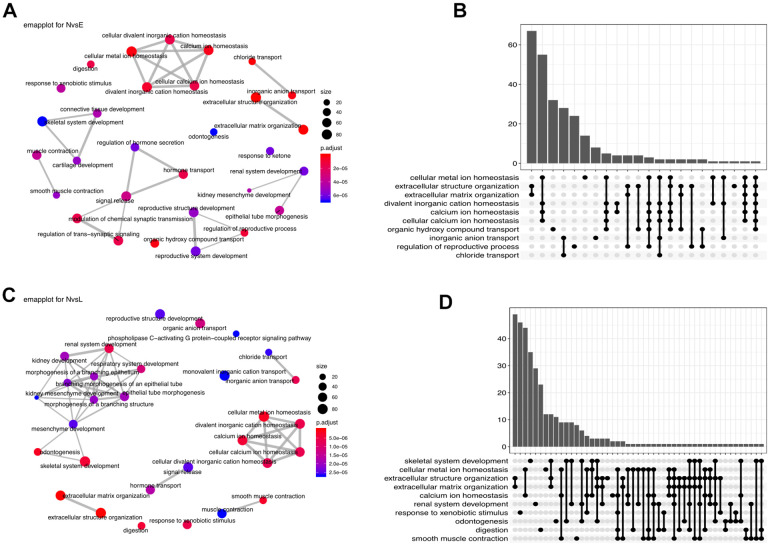
**Functional enrichment analysis.** (**A**) Enrichment network (emapplot)of functions regulated by the NvsE DEGs. (**B**) Upset plot of the top 10 NvsE-related functions. (**C**) Emapplot enrichment network of functions held by the NvsL DEGs. (**D**) Upset plot of the top 10 NvsL-associated functions.

To investigate the cellular functions regulated by the DEGs, we conducted Gene Ontology (GO) functional enrichment analysis (Figure 4).

Comparison between early stage and normal control samples revealed many interacting DEGs enriched mainly in homeostasis-related functions (Figure 4A, 4B). Between late stage patients and normal controls, the predominant DEG-enriched functional modules included ‘homeostasis’ and ‘multiple system development’ (Figure 4C, 4D). It implies that as the disease progresses, different biological functions are dynamically interfered.

### Stage-specific co-expression network analysis

**Figure 5:**
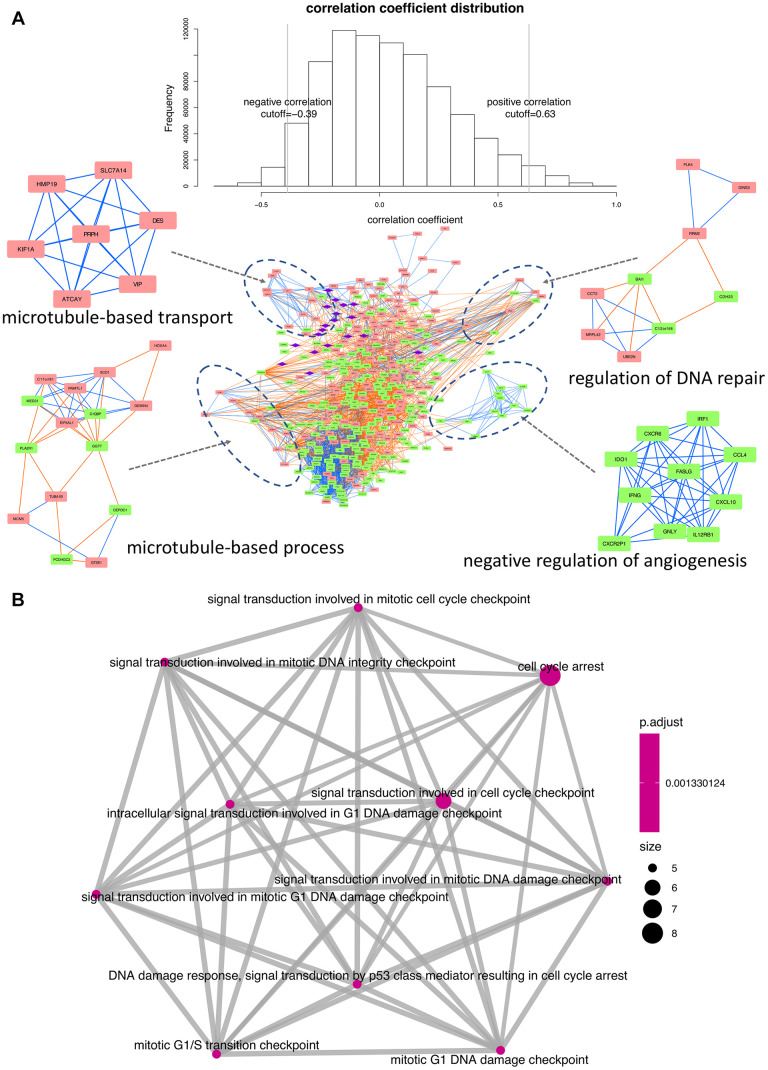
**Interaction network and functional enrichment analysis of DEGs and miRNAs between early and late stage.** (**A**) Co-expression network analysis. Purple diamonds represent miRNAs; red and green rectangles represent upregulated and downregulated DEGs, respectively. Orange and blue lines indicate, respectively, positive and negative correlations between nodes. Top-enriched functions are indicated under the corresponding modules. (**B**) Functional network depicting DEG-enriched processes regulated by the 19 differentially expressed miRNAs.

We next applied the Pearson’s correlation algorithm to assess potential regulatory influences exerted by differentially co-expressed miRNAs on the identified DEGs. As shown in Figure 5A, a total of 4,656 edges and 362 nodes were identified in the network, and four significant modules were extracted using the MCODE plugin.

The principal biological GO terms within the network were then obtained using the BiNGO plugin [11]. The process of ‘microtubule-based transport’ was seemingly activated, since all the involved genes were upregulated in late stage patients. In contrast, ‘negative regulation of angiogenesis’ was apparently inhibited, as all the corresponding regulatory components were downregulated. Lastly, significant enrichment in both upregulated and downregulated DEGs was detected for ‘regulation of DNA repair’ and ‘microtubule-based process’.

Functional enrichment analysis was also conducted to assess the biological roles of the 19 differentially expressed miRNAs in relation to their target genes (Supplementary Table 1). Results showed that 86 DEGs were targeted by these 19 miRNAs, exerting a predominant regulatory influence on cell cycle dynamics (Figure 5B).

### Recurrent somatic mutation selection

**Figure 6:**
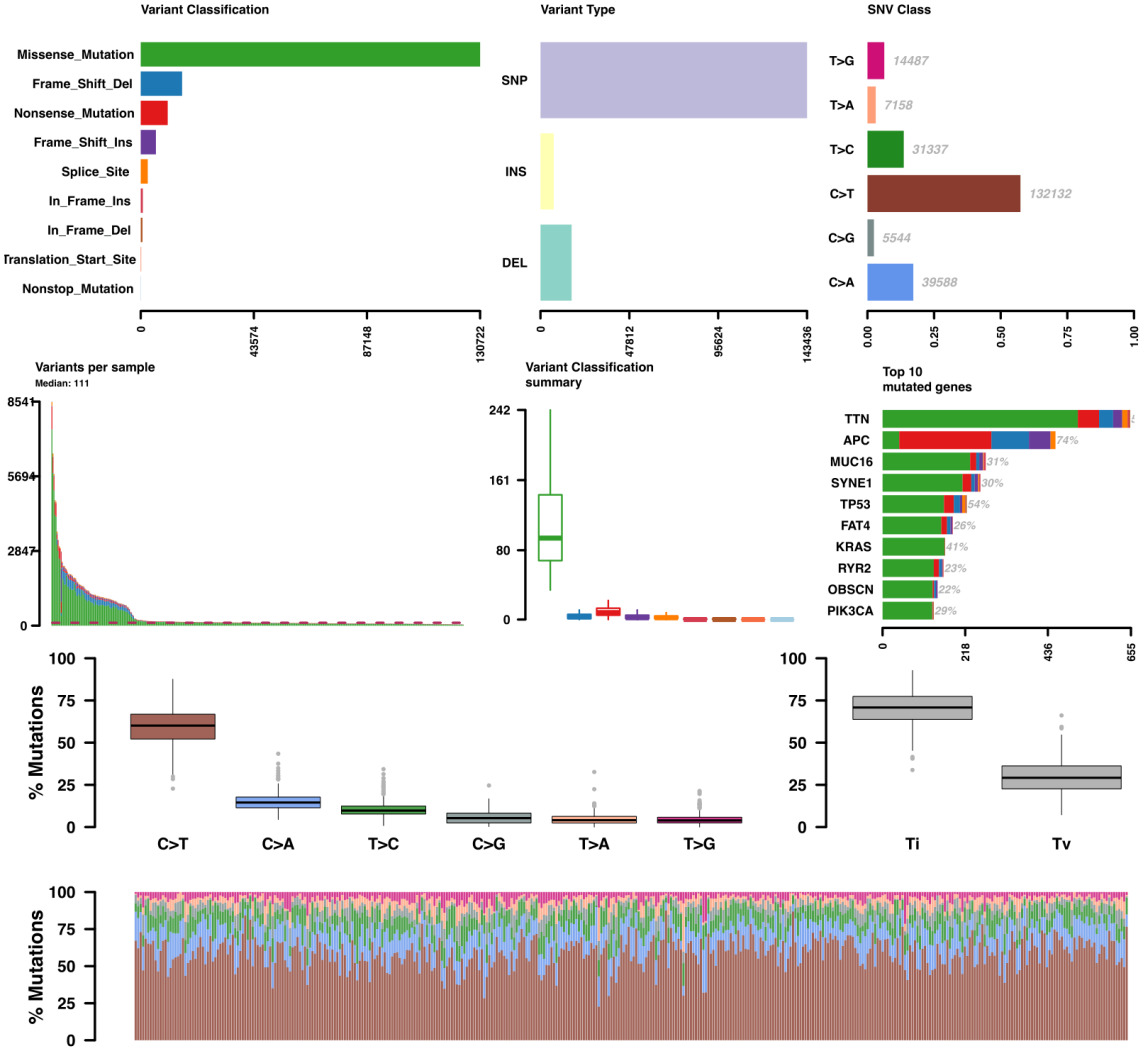
**Somatic variant analysis of COAD-TCGA data.** Variants per sample are shown as a stacked barplot and variant types as a boxplot summarized by variant classification.

A mutation profile analysis of WES data from COAD patients revealed that missense mutations were the most dominant variants (Figure 6). In turn, a transition (Ti)–transversion (Tv) bias was one of the significant features in both whole-genome sequencing (WGS) and WES data. In COAD-WES data, the C>T transition was the most distinct feature, which suggests an essential role for oxidative DNA damage in COAD pathogenesis [12].

On gene-level analysis, the top 10 mutated genes included *TTN, APC, MUC16, SYNE1, TP53, FAT4,* *KRAS, RYR2, OBSCN,* and *PIK3CA*. Then we investigated the recurrence of each mutation across patients from each stage. All recurrent somatic mutations with a frequency larger than 5% were selected.

### Identification of candidate neoantigen-coding genes

**Figure 7:**
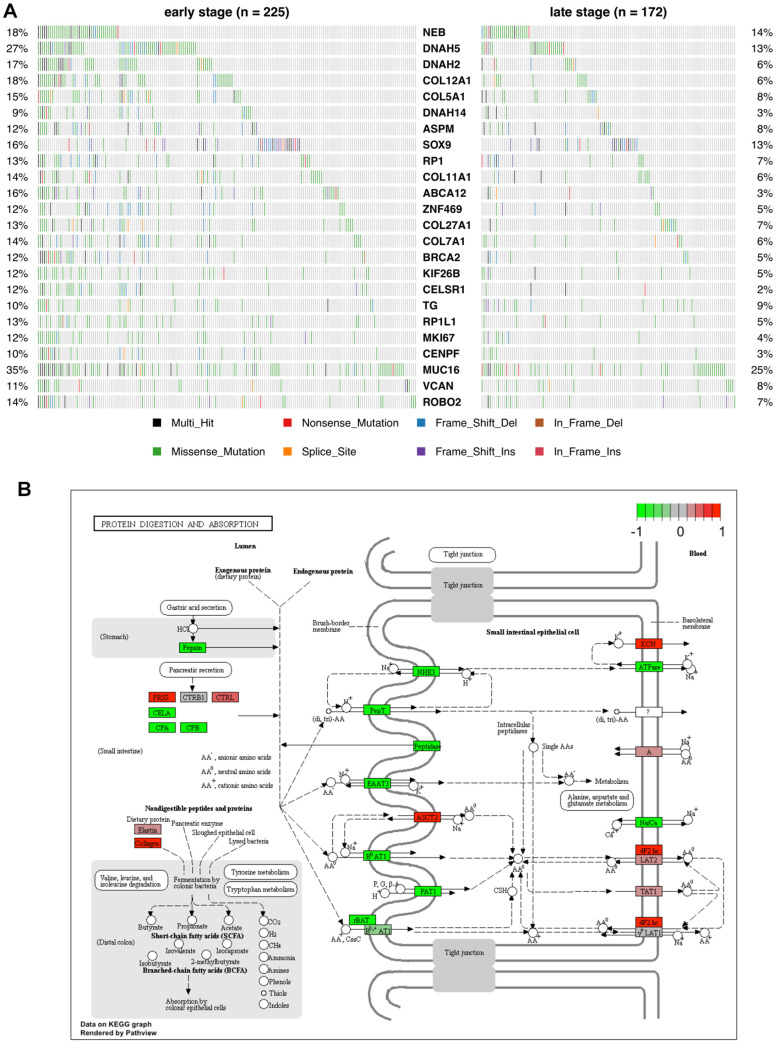
**Mutational profile and KEGG pathway analysis of candidate COAD neoantigen-related DEGs.** (**A**) Mutation frequency data. The percentage of patients harboring the color-coded variations listed at the bottom are indicated on the left and right sides. (**B**) KEGG pathways enriched in the 24 candidate neoantigen-related genes. Red and green boxes indicate up- and down-regulated genes, respectively.

Under the assumption that genes with one or more recurrent mutations potentially leading to neoantigen production are exclusively overexpressed in tumor tissues and not in normal ones, we selected the recurrent mutations within differentially overexpressed genes between early and late stage patients. We eventually identified 24 genes harboring recurrent somatic mutations in at least 5% of patients from either stage (Supplementary Tables 1, 2). The host genes and their corresponding mutational frequency in either stage are shown in Figure 7A.

As seen in Figure 7A, the host genes of the candidate neoantigens were different from the top-mutated genes identified by the canonical protocol. A main reason for this is that the most mutated genes might be either silent in tumor tissues or expressed at similar levels than normal ones. Among the 24 genes identified by our protocol, *NEB* and *DNAH5* were found to be mutually exclusive, especially in late-stage patients.

We next performed KEGG pathway enrichment analysis for the 24 genes to evaluate their functional properties (Figure 7B). Among them, 5 genes involved in collagen synthesis, i.e. *COL11A1, COL12A1, COL27A1, COL5A1,* and *COL7A1,* were enriched in the protein digestion and absorption pathway.

### Correlation of COAD-associated neoantigen genes with tumor infiltrating immune cells and predicted miRNAs

**Figure 8:**
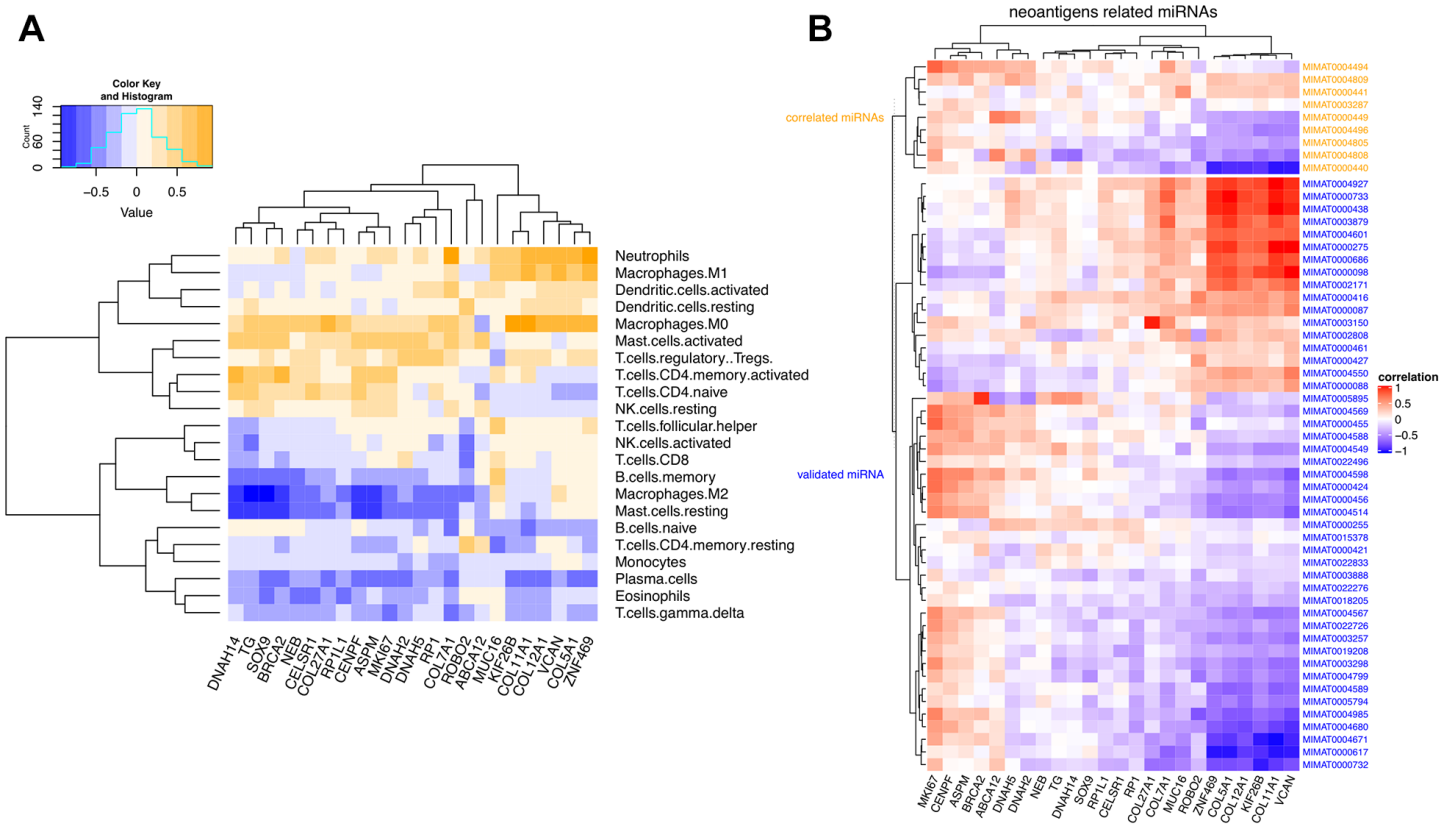
**Correlation matrix between neoantigen-associated DEGs and immune cells and miRNAs.** (**A**) Correlation between COAD-related neoantigen genes and immune infiltrating cells. (**B**) Correlation between COAD-related neoantigen genes and miRNAs. Validated and predicted miRNAs are marked in blue and orange, respectively.

The Pearson’s correlation coefficients between the 24 neoantigen-associated DEGs and COAD-infiltrating immune cells (determined by RNA-seq data) are shown in a heatmap on Figure 8A. Intuitively, we observed two correlation patterns within the 22 immune cells analyzed. The most prominent, positively correlated immune cells consisted of neutrophils, macrophages, dendritic cells, activated mast cells, and naïve and activated T cells. In contrast, B cells, resting mast cells, resting T cells, monocytes, plasma cells, and eosinophils were mainly negatively correlated with the candidate neoantigen-forming DEGs.

We further investigated the correlation between the 24 selected neoantigen genes and miRNAs. We combined 47 validated miRNAs from three miRNA databases and 9 predicted miRNAs based on expressional correlation. These 9 predicted miRNAs were all correlated with at least one of the 24 neoantigen-related host genes. A correlation matrix between the 24 neoantigen genes and the 56 miRNAs is shown in Figure 8B. It can be seen that six host genes (*ZNF469, COL5A1, COL12A1, KIF26B, COL11A1,* and *VCAN*) were the most significantly regulated by the overall miRNA pool, especially by MIMAT0000440.

Although similar correlation patterns were observed for both predicted and validated miRNAs, stronger links were identified for the 9 predicted miRNAs. This suggests their potential involvement in the regulation of COAD progression.

### Diagnostic model construction

**Figure 9:**
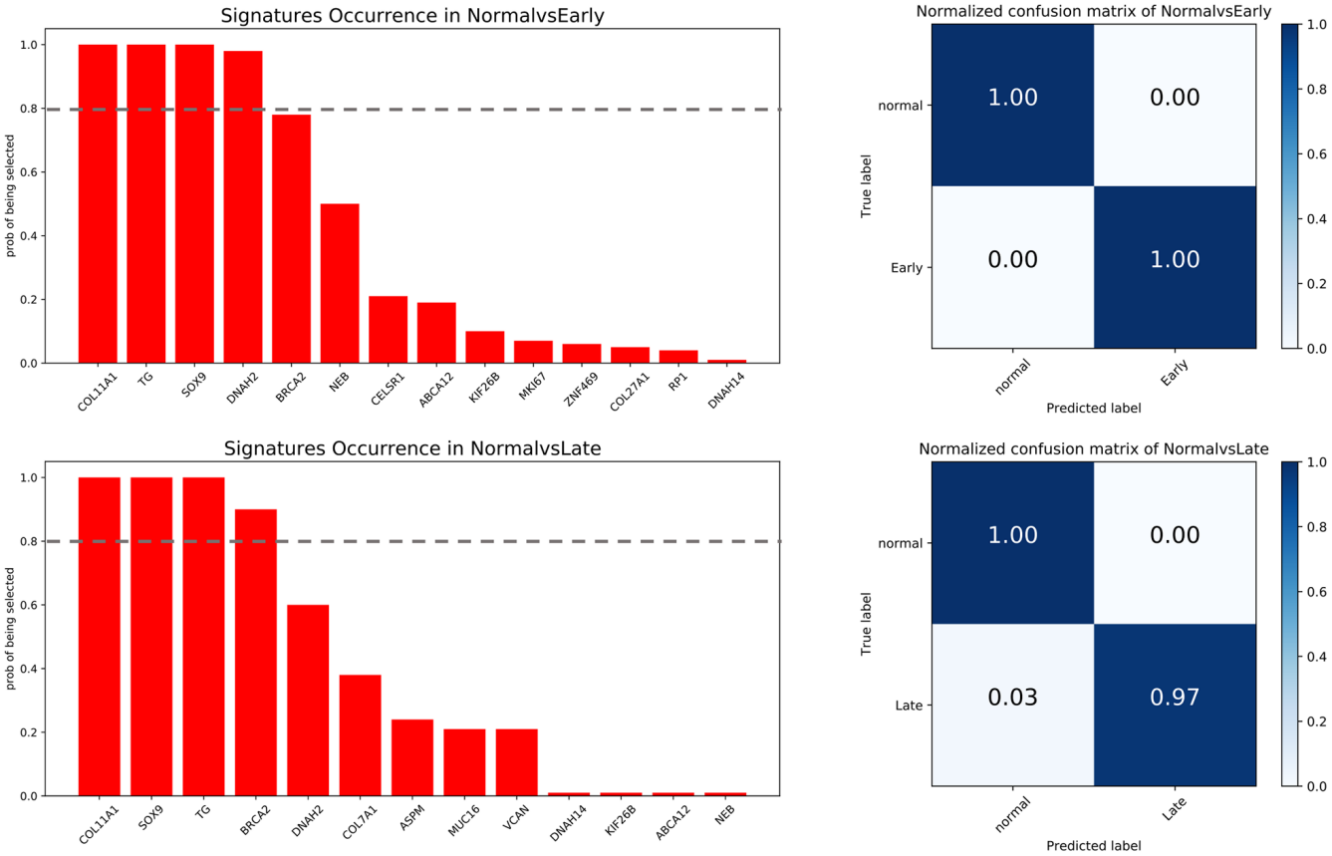
**Feature selection and confusion matrices.** Top: normal vs early stage. Bottom: normal vs late stage. The x-axis of barplot graphs lists featured genes and the y-axis indicates how many times each feature is selected over 100 permutations. The grey dash line represents the significance cutoff (0.8). The x-axis in the confusion matrices represents the predicted labels and the y-axis represents the true labels.

Since the candidate neoantigen-forming DEGs were all overexpressed and tumor-specific, we evaluated their potential as diagnostic markers. Applying the random forest algorithm, we obtained four early stage-related signatures, represented by *COL11A1, TG, SOX9*, and *DNAH2*. Likewise, four late stage-related signatures, namely *COL11A1, SOX9, TG*, and *BRCA2*, were also selected (Figure 9). Except for *DNAH2* and *BRCA2*, all the other genes were shared by the two tumor stages. This suggests a relatively stable expression for these DEGs during COAD progression. However, the stage-specific signatures indicate that some differences exist between the two stages.

Using the selected genes as signatures, the trained predictor was tested against a validation dataset. The accuracy of predicting early and late stage cancer was 100% and 97%, respectively. These data suggest that the neoantigen-related DEGs identified by our protocol have the potential to be exploited as both therapeutic targets and diagnostic biomarkers.

### Evaluation of neoantigen-related DEGs as survival indicators in COAD

**Figure 10:**
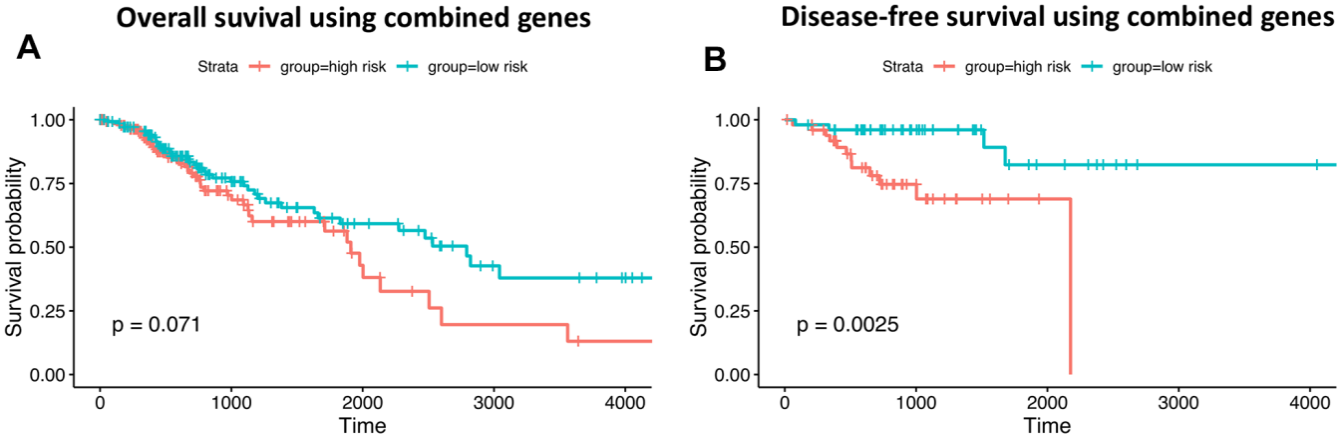
**Prognostic ability of neoantigen-related gene signatures in COAD.** (**A**) Overall survival. (**B**) Disease-free survival. Time is expressed as days in the graphs’ x-axis.

The potential impact of the 24 neoantigen-associated DEGs on survival prognosis was investigated using stepwise regression. Eight genes, i.e. *ZNF469, VCAN, RP1, MUC16, KIF26B, COL5A1, COL12A1*, and *CENPF*, were chosen to build a signature for overall survival (OS). In turn, another 8-gene set, including *RP1L1, VCAN, SOX9, KIF26B, MUC16, COL5A1, COL11A1,* and *CELSR1*, was selected as a relevant prognostic signature for disease-free survival (DFS). Based on expression data for these signature genes, high- and low-risk patient groups were determined by the regression model prior to construction of Kaplan-Meier curves comparing OS and DFS (Figure 10A, 10B).

Despite a non-significant log-rank P value for OS (0.07), the 8-gene set still has valuable clinical implications. That the P-value did not reach statistical significance is attributable to the mixture of patients surviving no more than 3 years. However, the survival difference is obvious for patients surviving longer than 3 years. Moreover, another 8-gene set was significantly predictive of DFS (P-value=0.0025). These results show that differential expression of signature genes can be used to successfully predict OS and DFS among high- and low-risk patients.

### Comparative analysis of neoantigen-related DEG expression between COAD and multiple cancers

**Figure 11:**
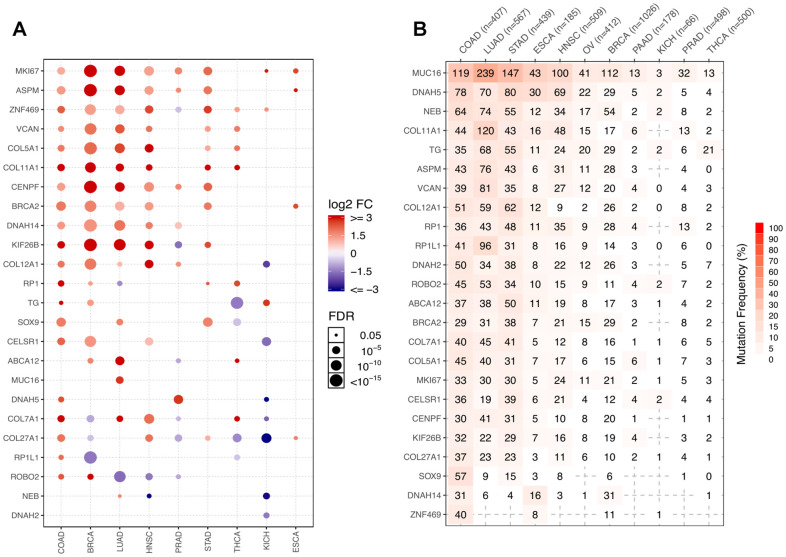
**Representation of the 24 COAD-related neoantigen genes in other cancers.** (**A**) Comparative expression analysis of the 24 host genes across 9 cancers. (**B**) Number of mutated samples across 11 cancers.

To assess whether the 24 overexpressed host genes harboring recurrent mutations associated with candidate neoantigens identified herein were specific to COAD, we evaluated their expression in 10 other cancers (Figure 11).

Genes with FC > 2 and FDR > 0.05 were retained for analysis. Normalized RSEM values were used as expression values in GSCALite, but log2(x+1) transformed RSEM normalized count was used in our RNAseq analysis. Because of the different quantification methods, some slightly overexpressed genes, such as *NEB* and *DNAH2*, whose log2FC values were respectively 2.2 and 2.6 in NvsT analysis, were missed by the GSCALite analysis.

We found that most host genes were overexpressed in multiple cancers besides COAD, including *BRCA, LUAD, HNSC*, and *STAD*. Other genes, including *DNAH5, COL7A1, COL27A1, RP1L1*, and *ROBO2*, seemed instead to be COAD-specific, as they were silent or even downregulated in other cancers (Figure 11A). Most of these genes were however recurrently mutated in COAD and other digestive system cancers such as stomach adenocarcinoma (STAD) and esophageal carcinoma (ESCA). They were also highly mutated in lung adenocarcinoma (LUAD), but this may be attributed to the large number of mutations characteristic to this entity (Figure 11B).

**Figure 12:**
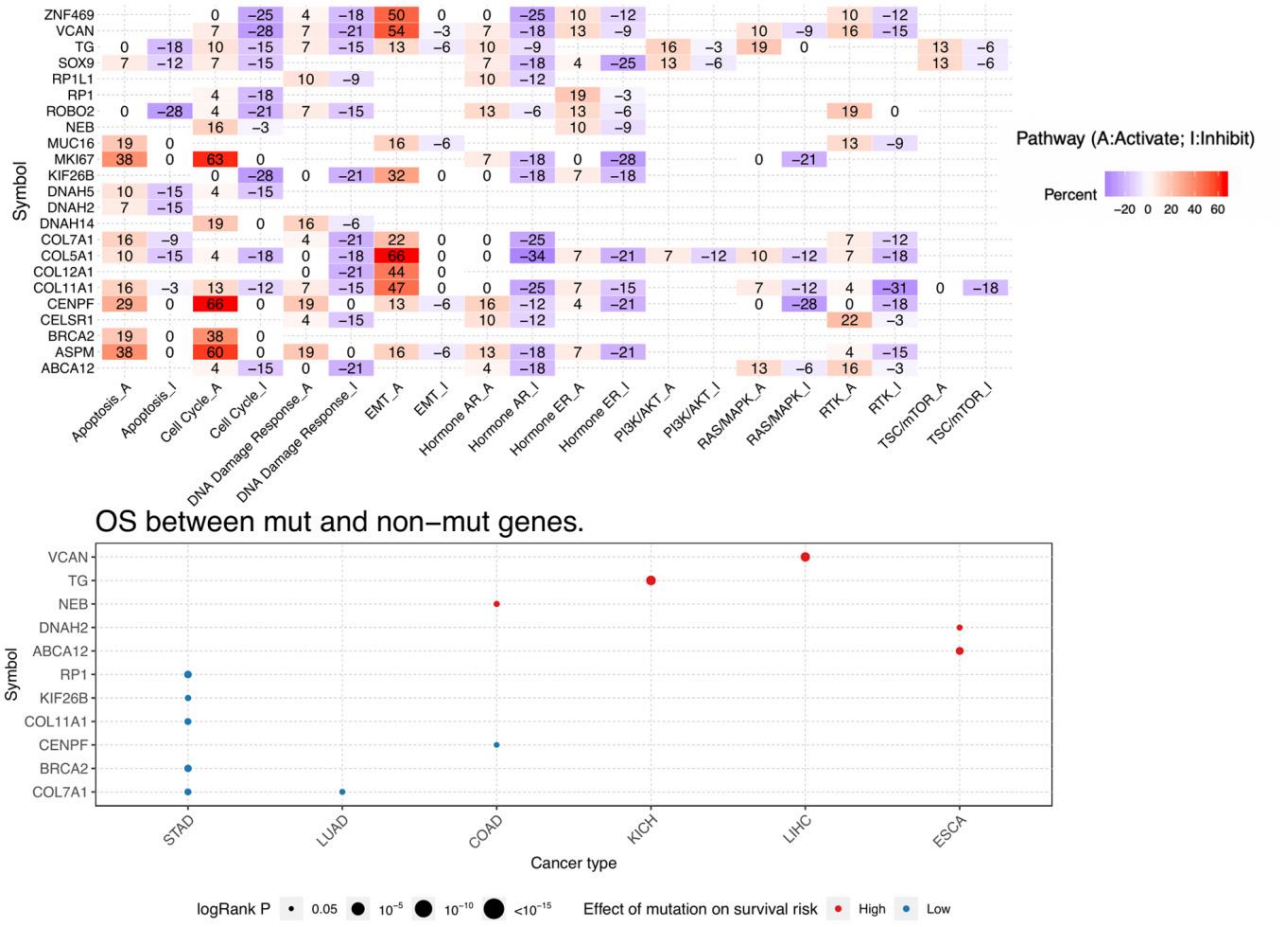
**Pathway activity and mutation survival analysis.** Top: Inferred activity of the identified host genes in biological pathways. Red and blue represent percent activation or inhibition. Bottom: Relationship between mutations in identified DEGs and survival prognosis for selected cancers.

Pathway analysis indicated that some biological functions were significantly impacted by mutations in the host genes analyzed. For instance, apoptosis, cell cycle, DNA damage response, EMT, and hormone_AR/ER were all simultaneously or alternatively activated and/or inhibited by the different mutations (Figure 12).

## DISCUSSION

Immunotherapy approaches have gained prominence in the treatment of many cancers, especially leukemia, lymphoma and lung, kidney, and bladder cancer. Seven standard treatments, including surgery, radiofrequency ablation, cryosurgery, chemotherapy, radiation, target therapy, and immunotherapy, are currently used for COAD. Resection and anastomosis are the major strategies for early stage (stage I and II) colon cancer patients, while chemotherapy may be further indicated for stage III patients. Immunotherapy is currently used to treat stage IV and recurrent colon cancer patients [13], and its potential use in early stage patients remains controversial. This is largely due to uncertainty about both the mutational changes impacting early to late stage progression, as well as the immune influences that shape this transition.

Tumor neoantigens are modified proteins expressed on the surface of tumor cells and recognized as “non-self” or foreign by cells of the immune system [14]. Generally, these foreign proteins derive from tumor-specific mutations that change the original peptide sequence and/or structure. Tumor neoantigens have attracted a great deal of attention as potential targets for immunotherapy, including individualized or broad-spectrum cancer vaccines. To reduce the risk of potentially severe autoimmune reactions, proper screening protocols are required to identify clinically actionable tumor neoantigens. Ideally, the mutations that give rise to neoantigens should be recurrently observed in a significant fraction of patients so that the therapy can be broadly applied. High-throughput sequencing technologies provide the opportunity for large-scale screening of potential neoantigens in cancer patients. For instance, approaches using WES data alone or together with gene expression data have been used in clinical trials of checkpoint inhibitors [7].

In this study, we integrated WES and RNA-Seq data to screen candidate neoantigen-hosting genes in colon cancer. After selecting stage-specific DEGs through RNA-Seq analysis, recurrent mutations in the overexpressed genes were further selected through WES analysis. Based on mutational recurrence rates among COAD specimens, we then applied the random forest, a supervised machine learning model, to construct two gene signatures that showed high diagnostic accuracy to discriminate early and late tumor stages. In turn, survival analyses showed prognostic differences between patients with and without these recurrent mutations.

We identified *SOX9* and *COL11A1* as relevant biomarkers of diagnosis and survival prognosis in COAD. *COL11A1* expression is upregulated in many cancers, including colorectal, breast, and ovarian cancer, and head and neck squamous cell carcinoma [15–19]. Previous studies suggested that *COL11A1* regulates tumor progression through the APC/beta-catenin pathway, and inhibits apoptosis by modulating the NFkB pathway [20, 21].

Elevated *SOX9* expression was characteristic of COAD and this gene was thus included in the diagnostic signatures for early and late stage tumors. *SOX9* overexpression is associated with increased mortality in many cancers, including colon and rectal cancers [22–24]. *SOX9* is involved in multiple functions that promote cancer progression, such as proliferation and transformation, and resistance to apoptosis and chemotherapy [23, 25].

Among the 24 neoantigen-related genes identified herein, *COL11A1*, *COL12A1, COL27A1, COL5A1*, and *COL7A1* participate in the synthesis of various collagen types. Excessive, abnormal deposition of collagen chains may lead to enhanced activation of fermentation by colonic bacteria. This phenomenon has been linked to colon carcinogenesis; as colon cancer progresses, the activity of colonic microorganisms becomes more intense, which would intensify the digestion and absorption of proteins [26–29]. Other markers identified by us as candidate host genes for neoantigens have already been postulated as driving factors in COAD. These include *BRCA2* [30], *MKI67* [31], *MUC16* [32], *RP1* [33], and *VCAN* [34].

Novel findings of our study include the new diagnostic and prognostic signatures, the observed correlation between the neoantigen-associated genes and specific tumor-infiltrating immune cell populations, and the predicted regulatory influences exerted on the former by several miRNAs. Moreover, we identified several DEGs that had not so far been associated with COAD. These include *NEB*, which predicted high survival risk in COAD patients, and *DNAH2* and *ABCA12*, which are considered essential prognostic indicators for ESCA. Two other DEGs, *CENPF* and *CELSR1*, were in turn selected in this study as prognostic indicators of OS and DFS in COAD patients.

*ABCA12* is a member of the ATP-binding cassette (*ABC*) family of transporters, which are essential mediators of chemoresistance [35]. *DNAH2* encodes for the dynein heavy chain, which also has ATPase activity [36]. *CENPF* is a widely studied driver gene in multiple cancers, such as gastric [37], prostate [38], breast [39], and bladder [40] cancer. Functional experiments revealed positive effects of *CENPF* on cellular proliferation, migration, and invasion [41]. *CELSR1* was shown to promote progression and paclitaxel resistance of ovarian cancer *in vitro* and *in vivo* [42]. Our data thus reveal novel associations between these genes and COAD, some of which present potential relevance as diagnostic/prognostic indicators.

Somatic mutations may lead to generation of neoantigens for T-cell recognition, leading in turn to increased recruitment of various kinds of immune cells [5]. Therefore, uncovering the relationship between tumor neoantigens and infiltrating immune cell populations will greatly boost the efficacy of immunotherapy [43–45]. Correlation analysis between COAD-associated neoantigen genes and tumor-infiltrating immune cells indicated that as neoantigen expression increases, the fractions of several immune cell types rise accordingly. Specifically, our data showed that some immune cells, such as dendritic cells, mast cells, and T cells were overrepresented and synchronously activated in samples with high neoantigen expression. Meanwhile, other immune cells, such as B cells, monocytes, plasma cells, and eosinophils, were inhibited and downregulated. This suggests an obvious link between tumor neoantigen expression and differential representation and activity of tumor-associated immune cell populations. Although further research is warranted, this suggests the possibility of assessing neoantigen levels to estimate the immune status of tumors.

To gain insight on the regulatory landscape of the 24 DEGs harboring potential COAD neoantigens, we correlated their expression with that of miRNAs. Nine non-validated miRNAs were predicted to regulate these 24 neoantigens. The candidate neoantigen genes with the strongest negative correlation with these miRNAs were *ZNF469, COL5A1, COL12A1, KIF26B, COL11A1* and *VCAN*. Although experimental confirmation of these interactions is lacking, these miRNAs correlated with at least one of the genes more strongly than any of the validated miRNAs.

The recurrent mutations identified herein are predominantly observed in COAD, compared to other cancers. ESCA and STAD are the cancers most closely related to COAD in terms of recurrent mutation profiles. This is not unexpected, because these three cancers arise in the digestive system and share a similar tumor microenvironment [46]. However, the predicted impact of the identified mutations on the survival risk of patients was rather dissimilar among these entities. For instance, our analyses showed that mutations in *NEB* correlate with higher survival risk in COAD patients, but have no obvious impact on ESCA patients. Conversely, mutations in *DNAH2* and *ABCA12* were associated with higher risk in ESCA, but not COAD, patients. In turn, mutations in *RP1, KIF26B, COL11A1, BRCA2,* and *COL7A1* had no prognostic significance in COAD patients, but correlated with lower risk in STAD patients. Besides ESCA and STAD, we found that LUAD also shares a similar mutational profile as COAD. A possible reason for this is that LUAD patients tend to harbor more mutations due to exposures such as tobacco smoking [47].

In summary, our study integrated transcriptome and whole-exome sequencing data from COAD-TCGA and identified 24 DEGs harboring recurrent somatic mutations with neoantigen-forming potential in COAD patients. Among these candidate neoantigen genes, *NEB, DNAH2, ABCA12, CENPF,* and *CELSR1* were newly identified as COAD biomarkers, while *DNAH5, COL7A1, COL27A1, RP1L1,* and *ROBO2* had mutational profiles specific to COAD, compared to other solid tumors. We further constructed two diagnostic signatures, composed respectively of 4 early stage-related genes (*COL11A1, TG, SOX9,* and *DNAH2*) and 4 late stage-related genes (*COL11A1, SOX9, TG,* and *BRCA2*), which predicted COAD stage with high accuracy. Furthermore, several candidate neoantigen-yielding genes identified herein showed significant correlations with both miRNAs and diverse tumor-infiltrating immune cell types, and therefore represent promising therapeutic targets for immunotherapy. Nevertheless, further research is warranted to experimentally validate the association between the recurrently mutated DEGS identified herein and the generation of tumor-specific neoantigens, and to explore the functional impact of the identified mutations on tumor biology.

## MATERIALS AND METHODS

### Data collection

We downloaded WES and RNAseq data of COAD from the TCGA database (https://www.cancer.gov/tcga). We also retrieved the clinical information for all patients, including MNT stage and survival data. A total of 459 COAD cases (329 with transcriptome and 399 with exome sequencing data, respectively) were thus obtained. We also retrieved the miRNA sequencing data for 261 samples, involving 2,113 microRNAs. Gene expression profiles for all patients were determined using the Illumina HiSeq 2000 RNA Sequencing platform. Level 3 data were downloaded from TCGA data coordination center. This dataset shows the gene-level transcription estimates as the log2(x+1) transformed RSEM-normalized count. Patients diagnosed with tumor stage I/II were assigned to the early stage group, and those with more advanced stages were assigned to the late stage group.

### Differential gene expression analysis

We analyzed RNAseq data from 329 COAD patients, 41 of which had matching data for normal tissues. The limma algorithm [48] was used to identify differentially expressed genes (DEGs) in early and late stage COAD samples, compared with normal tissue specimens. As the microRNA data was only used to explore potential regulatory actions on protein-coding genes, differential microRNA expression was only assessed between the two tumor stages. All genes and microRNAs with P < 0.05 and logFC values over the 95% confidence limit were considered as differentially expressed.

### Gene clustering and functional analysis

Overexpressed genes in either stage were selected to establish candidate neoantigen pools. These genes were either lowly expressed or silent in normal tissues, and therefore potentially good targets to avoid adverse effects if used to develop targeted therapies [49]. Hierarchical clustering [50] was used to visualize gene expression patterns in normal and tumor specimens. ClusterProfiler and enrichplot R packages [51] were used to conduct functional enrichment analyses.

### Co-expression network analysis

DEGs and miRNAs identified at early and late tumor stages were used to construct the co-expression network [52]. Transcript (mRNA or microRNA) co-expression was determined by Pearson’s correlation analysis [53], with a correlation coefficient cutoff determined based on 95% CIs for all pairs. The network was constructed using Cytoscape 3.8.0 software [54]. Significant modules were mined from the network using the MCODE plugin with default parameters [55].

### Recurrent somatic mutation selection

MAF files including somatic mutation information for exome sequencing data were retrieved from the TCGA database. We focused on nucleotide resolution and selected recurrent somatic mutations (i.e. those carried by at least 5% of patients), which represent potential therapeutic targets [56].

### Selection of candidate neoantigen-associated genes

Candidate neoantigen-forming genes were initially selected based on high expression in COAD samples and low or no expression in normal ones [57]. Among those, we selected the early and late stage genes that harbored somatic mutations that were recurrent in at least 5% of COAD specimens. Putative neoantigen genes corresponding to each stage were compared to determine stage-specific differences with potential correlation with tumor progression.

### Analysis of tumor-infiltrating immune cell populations

We applied CIBERSORT [58], a computational method for quantifying immune cell fractions from RNAseq data, to evaluate infiltration rates for 22 immune cell types. Pearson’s correlation coefficients were subsequently computed to assess potential correlations between neoantigen genes and infiltrating immune cells. The correlation matrix was visualized by heatmap using the heatmap.2 R package.

### Prediction of miRNAs targeting candidate neoantigen-associated transcripts

The co-expression network described above was used to extract all the miRNAs correlated with at least one of the selected host genes of the candidate neoantigens. The correlated miRNAs included both validated (retrieved from miRecords [59], miRTarBase [60], and TarBase [61] databases) as well as unannotated transcripts. Then, we inferred potential miRNAs targeting the candidate neoantigens by cross-assessment with the validated miRNAs.

### Diagnostic model construction

To investigate whether the host genes encoding putative COAD neoantigens could serve as diagnostic signatures, we trained a random forest model as a diagnostic predictor [62]. The host genes corresponding to each stage were used as signatures. The whole data was randomly split into discovery (70%) and validation (30%) samples. For feature selection, we first randomly split the discovery data into train and test sets. A linear SVC model was used to select the most significant features [63]. This process was repeated 100 times, and the features selected in at least 80 iterations were considered as robust signatures. The predictor was trained with default parameters using the training set, and 10-fold cross-validation was used to evaluate the performance of the predictor [64]. Eventually, we applied the predictor to the test set and assessed the model’s accuracy.

### Survival analysis

The impact of candidate host genes harboring neoantigen-related mutations on COAD prognosis was initially assessed using stepwise regression [65]. Then, patients were separated into low- and high-risk cohorts based on individual expression frequencies. Survival analysis was conducted using Kaplan-Meier curves generated through the survival and ggsurvplot R packages [66, 67].

### Comparative neoantigen expression analysis

To investigate whether the neoantigen-related DEGs identified herein are COAD-specific or are also shared by other types of cancers, we used the GSCALite tool [68] to compare the corresponding expression patterns in datasets from ten additional cancers retrieved from the TCGA database.

## Supplementary Material

Supplementary Table 1

Supplementary Table 2
